# Executive Function Touch battery: Translation and preliminary measure validation for Pakistani preschoolers

**DOI:** 10.1371/journal.pone.0274431

**Published:** 2022-09-15

**Authors:** Hafsa Khalil Toor, Rubina Hanif

**Affiliations:** 1 Department of Psychology, Foundation University Islamabad, Islamabad, Pakistan; 2 National Institute of Psychology, Quaid-i-Azam University Islamabad, Islamabad, Pakistan; Leiden University: Universiteit Leiden, NETHERLANDS

## Abstract

Keeping in mind the importance of measuring early executive function (EF) skills in low and middle-income countries, the present study examined the feasibility and preliminary psychometric properties of a performance-based computerized EF measure; EF Touch, to be used with Pakistani preschoolers. Review of the content and Urdu translation of verbal instruction EF Touch battery was carried out by subject matter experts before data collection from the 120 preschoolers aged between 3.1 to 5.9 years. The feasibility report indicated that between 79.2% -100% of the preschoolers completed each executive function task. Confirmatory factor analysis revealed the unidimensionality of the EF battery. Item response theory models were used for the initial assessment of tasks and item parameters. Results demonstrated that each task worked invariantly across subgroups of preschoolers residing in low and middle-income households. Moreover, preschoolers showed differences on each task, and task scores reflect their latent EF skills in the low to moderate range. The battery was demonstrated as a feasible and reliable measure for use with low and middle-income countries specifically in Pakistan.

## Introduction

Executive function (EF) is considered to be a core ability of child development. Broadly defined, EF is an umbrella term that includes a set of higher-order cognitive skills that govern goal-directed behavior to novel or complex situations that contribute to problem-solving, planning, and decision making [1]. EFs are a set of skills a child use in everyday to learn, manage everyday life, to control thoughts, emotions, or behavior. There have been a range of researches suggested that there are three crucial EF components that include working memory, inhibitory control, and attention shifting that delineated during the preschool age as three distinct factors defining the construct of EF [1, 2]. Working memory is defined as the ability to retain and manipulate information for a short time. Inhibitory control involves the ability to control and resist impulsive actions or responses when engaged in the completion of a given task. Attention shifting refers to the ability to shift attention or cognitive set in response to the demand and priorities of the situation.

Generally, the development of EF is conceptualized within the biological framework, the development of the prefrontal cortex is considered as neural substrate for the development of EF [3, 4]. Extensive research evidence supports that EF starts to develop early during the preschool years until adolescence [1, 5]. These executive functioning skills in early childhood are linked to several positive outcomes in different domains e.g. school success and school readiness [6–8] social-emotional skills [9], and quality of life [10].

Evidence from research [11] has reported that environmental input is crucial for the development of EF skills and any environmental adversity can hinder the positive development of these skills. Conversely, positive input, specifically, the child’s socioeconomic environment is predictive of EF skills [12, 13]. However, most of the work on early EF conducted so far on the children who reside in high household income countries. There is a scarcity of research work on early EF, based on children who reside in low and middle-income countries (LMIC) where children are exposed to various adversities. Taking this into account, the present study aimed to bridge the gap, by assessing the feasibility and initial psychometric properties of performance-based EF measure.

Recent studies [14, 15] suggested that EF is a domain-general that is assessment procedure that are developed and are used in high-income countries can be used and validated in LMIC. Further, it suggested that significant advancement has been made in neurodevelopment assessment, specifically, EF skills in children in LMIC during the past few years nevertheless but there need for research that improve the development, evaluation, adaptation, and validation of current assessment procedures. The recent studies in Kenya and Pakistan [15–17] demonstrated the viability of performance-based EF assessment procedures for early childhood that were developed in high-income countries to be used and generalizable in LMIC, nevertheless, these studies lack and suggested the use of modern testing theory to analyze EF and assessment of uniformity of item parameters to a subgroup of the population.

The assessment tools that were used previously in LMIC used paper-pencil assessment procedure, performance-based assessment or battery [e.g. 16, 18], rating scale [e.g. 19, 20] that is to be completed by the parent, teacher, or caregiver of the child. These tests require proper training for the administrator and time to administer. The present study aimed to add to researches conducted so far on assessment procedures that measure EF in early childhood of children residing in LMIC using computerize based data collection procedure.

The objective of the present study was the initial validation of the EF Touch battery [21] for Pakistani preschoolers from lower and middle household income. The original administration of the EF Touch battery involved paper-pencil-based flipbooks, to use in early childhood for high-income countries; that method used to require multiple assessors to administer and record the scores of the child. Later the battery was adapted to Microsoft window-based computerize version which requires the use of external touch screen monitor and a laptop; where the preschooler or the child respond via external touch screen and the task instruction are to read by the assessor from the external laptop. To fulfill the objective, primarily, the study aimed for translation of the assessor task instructions and review for the cultural relevance of the task of the battery. Further, the study assessed the feasibility of a computerized EF battery to use with Pakistani preschoolers. Moreover, the study intended for the dimensionality of the complete battery and preliminary evaluation of properties of EF touch battery on a small Pakistani sample.

### Executive Function Touch

The Executive Function Touch [21] is a computerized version of a widely used battery of EF tasks, that have been developed and extensively used with children in early childhood (3–6 years); that primarily used paper-pencil format-based flipbooks [22, 23]. As mentioned earlier the battery was later on adapted to Microsoft Windows operating system and lately improve the standardization, efficiency, and sensitivity of the EF tasks [24]. Lately, the battery is adapted to the Android version using Tangerine™ software [15]. For the present study, the MS Windows version of the battery is used.

The EF touch battery comprises seven tasks and that measures three core components of EF i.e. working memory, inhibition control, and attention shifting of the preschoolers. It also includes one simple reaction‐time task. The battery requires one standard monitor that displays scripted instructions for the assessor to read to the child and a touch screen monitor that records the child’s response. The assessor may use a keyboard or mouse to controls the tasks of the battery. Each task of the battery takes approximately 5–7 minutes to complete and around 45–60 minutes for complete administration of the battery. Each task begins with an item demonstration, which is followed by a training session. The child is presented with the test items if they pass the training items. The passing of the training item is determined automatically by the programmed software failure of the task was defined as the failure to pass the training in two attempts. The battery is flexible that any task can be administered in any desired order. No task-specific material is required except for some configurations of monitors that are used to run the tasks of the battery. The assessor requires nominal training; the scripted instructions of each task facilities the assessor to administer the task effectively. A brief description of each task of the battery is in the method section.

The primary objective of the study was to translate and evaluate the psychometric properties of the EF battery; after following the standard procedure of translation, initially, Confirmatory Factor Analysis (CFA) was used to evaluate the dimensionality of each task. Further, Item Response Theory (IRT) models were utilized to each task to investigate whether each task and item worked consistently for children residing in the low and middle-income household using Differential Item Functioning (DIF). Moreover, the Test Information provided the reliability or accuracy of each item and task over the range of its latent ability (theta).

## Method

### Procedure

After the approval of the Institutional Review Board and Ethical Board of the institute, the present study followed three phases. Preschoolers’ assent, parents’, and authorities’ written consent for their child participation were obtained before initiation of any data collection procedures. Initially, the EF Touch battery was reviewed by experts and the preschoolers, in the second phase it was translated using a standardized procedure, and in the last phase, the data was collected from the Pakistani preschoolers.

#### Phase I: Review of the content of Executive Function Touch

To review the tasks of EF touch battery, for cultural relevance and appropriateness of the battery to use with preschoolers two exclusive reviews were carried out. First, a review session was conducted with 20 preschoolers; ages ranged 3 to 5 years; were contacted in schools and homes. The main objective of this session was to assess whether the preschoolers could understand and identify the items and pictures of EF touch battery, moreover, to evaluate if the tasks were age appropriate for investigation of EF. After rapport building preschoolers were presented with different pictures of colors, symbols, sound, and animals, that were present in the battery. Second, the content review of the EF touch battery tasks was carried out with experts; with extensive experience in early childhood assessment and research in Pakistan. The content review sessions suggested that the task stimuli, verbal instructions, and general assessment approach of EF tasks were culturally appropriate for Pakistani preschoolers and recommended the need to translate the English instructions of EF touch in the Urdu language.

#### Phase II: Translation of verbal instruction of Executive Function Touch

The translation of the verbal instruction of the EF touch battery was carried out using the forward and backward method of the Brislin model of cross-cultural translation [25]. The English verbal instruction of each task was translated into Urdu by expert translators and the Urdu version was back-translated and compared back into the English version by expert suggested by the Brislin method. After the verbal instructions were finalized, the EF touch battery was ready to be administering for preliminary testing with Pakistani preschoolers.

#### Phase III: Data collection

At first, public and private schools from the city of Rawalpindi and Islamabad were approached to research their respective schools. The preschoolers of the selected schools represented low and middle-income household families. Minimum two visits were paid at the schools before the assessment of EF skills. The school authorities were given a brief overview regarding the objective, procedure and ethical concerns, and confidentiality of the data for the present study. After the grant of permission from the parents and school authorities, the basic demographic information about each preschooler was obtained from the school and teachers. After rapport building, each preschooler was assessed individually in a quiet space in their school on EF touch battery. Administration of all the tasks of the battery was in the national language Urdu. For the present study data was collected using MS windows version, it included the use of a laptop for the assessor to read instructions, and the preschooler responded through an external touch screen monitor. To create a more uniform assessment environment the monitor was placed in a position at the level; where the preschooler was comfortable to see and touch the monitor with ease.

### Participants

Initially, a total of 126 preschoolers were approached to participate in the current study, but six preschoolers either refused to participate or continue during the assessment. Therefore a total of 120 preschoolers (57 girls, 63 boys) participated in the present study from 24 different preschools of Rawalpindi/Islamabad city of Pakistan. The age range of preschoolers was 3.1 to 5.99 years (*M =* 4.60, *SD =* .90). From each school, the preschoolers were selected randomly, as mentioned earlier, consent from the parents was taken and only those preschoolers were selected who were willing to participate in the study. Preschoolers with premature birth or any physical or psychological illness reported by parents or preschool teachers were excluded from the sample. All the preschoolers in the sample were familiar with touch screen usage; at least one family member of each participant owned a touch screen gadget.

### Measure

#### Executive Function Touch

*EF Touch*: *Arrow*. Arrow is 36 items inhibitory control task, that measures child’s ability to control a dominant response. Two buttons appear on the left and right sides of the touch screen monitor. Preschooler follows the instruction of touching the button in which direction the arrow points. Three blocks of 12 arrows are presented. In the initial trials, in the first block, all the arrows appear above on the same side of the screen as the button (congruent condition). During later trials, the second block, all the arrow appears above on the opposite button to which they point (incongruent condition). In the third block, all the arrow appears in a combination of mixed location (mixed condition). Each item is presented for 3000 milliseconds. The task accuracy and reaction time of the response for each child is recorded for each item. Mean accuracy for the number of incongruent and mixed conditions is used as the performance of the preschooler.

*EF Touch*: *Silly Sound Game*. This task contains 17 items that measure the inhibitory control of the preschoolers. It is parallel to the Stroop task, in which a preschooler must inhibit a learned response. Each item is presented simultaneously with a picture of a cat and a dog; with random left or right order on the screen along with dog bark or cat meow sound. After the assessor makes sure that the preschooler can distinguish between the typical sounds made by each animal, they are instructed to play a silly game and require to touch the picture of the cat when they hear the bark of the dog and to touch the picture of the dog when they hear the meow of the cat. Each item is presented for 3000 milliseconds. The task accuracy and reaction time of the response for each child is recorded for each item. The mean accuracy of responses for all items is used to represent the performance of the preschooler.

*EF Touch*: *Houses*. Houses is 18 items task, which measures the working memory of the preschoolers. Each item is presented with pictures of one or more houses, each house contains a picture of an animal, a color (similar to a colored dot), or a colored animal in random order. Items are organized into a set of 2, 3, 4, and 5 houses. The preschooler is asked to label verbally the name of the animal, color in each house. After a brief period, the houses are presented again without any animal or color in them. The preschooler is then asked to recall one piece of information in each house i.e. either the animal or color of the animal. The task becomes difficult with each trial when the number of houses increases. Mean accuracy of responses of all items is used to represent the performance of the preschooler.

*EF Touch*: *Something’s the Same*. This is 30 items task, which measures attention shifting of the preschoolers. For 20 initial items, the preschooler is presented with two pictures e.g. two animals; two flowers, or two chairs, etc. that are similar concerning a single dimension of color, shape, or size. In the beginning, the preschooler is informed how two pictures are the same on some dimension. A third picture is then presented alongside the original two pictures and asked to select how new picture is similar to one of the two original pictures on some other dimensions (e.g., red chair and yellow chair are similar because they are chairs; the third new picture of red flower is similar to the red chair because they are both red). For last 10 items, the preschooler is instructed differently; they are presented with three pictures at once and asked to select both dimensions of similarity i.e. to identify and select pair of pictures that are similar along one dimension and then a select second pair of the picture on another dimension. The mean accuracy of responses for all items is used to represent the performance of the preschooler.

*EF Touch*: *Pig*. This is 40 items task, which measures inhibitory control. The preschooler is presented with a large green button on the screen, which pops up when it is touched. A single picture of different animals is displayed one by one, above the green button, and are instructed to pop the button every time they see an animal, (known as the *go* response) except when the picture of a pig is shown (known as a *no-go* response). Each item was displayed for 3000 milliseconds. The accuracy and reaction time of all the responses are recorded. Mean accuracy for the number of no-go responses is used as the performance of the preschooler.

*EF Touch*: *Pick the Picture*. This task contains 32 items, which measure the working memory of the preschooler. They are presented with series of sets of pictures, that varies and progress in number e.g. set of 2, 3, 4, 6 pictures. For each set of items, preschoolers are instructed to touch any picture of their choice. The same set of pictures is presented repeatedly, with the random change in the position of the pictures on the screen. Preschoolers are asked to continue choosing a new picture in the set so that "all the pictures get a turn". The mean accuracy of responses in each set of pictures is used as the performance of the preschooler.

*EF Touch*: *Farmer*. *This* is 36 items task, which measures spatial working memory. In this task, a 4 by 4 grid of squares are displayed on the screen, as a farmer’s field. The preschooler is shown one of the farmer’s animals in a sequence in the highlighted grinds (fields) and told that farmer’s animal is lost. The preschooler is instructed to help the farmer by touching the fields in the same order in which the animal walked in i.e. the order in which the animals were highlighted in the grids. They are instructed to fulfill the requirement of touching the necessary number of fields in which animals appeared and if they don’t remember, they use their best guess. The mean accuracy of responses for all items is used to represent the performance of the preschooler.

*EF Touch*: *Bubble*. This is 30 items task, which measures simple reaction time. Preschoolers are presented with several blue bubbles on the screen, one at a time. They are asked to pop up each bubble as fast as they can. Each bubble is presented for 5000 milliseconds. The reaction time was calculated from the onset of the bubble and the preschooler touch to popup. Mean reaction time for all accurate responded items is used as the processing speed or simple reaction time.

*EF Touch*: *Quality ratings of the tasks*. After completion of each task, the assessor gives a subjective evaluation of data quality on a single item which assesses the preschooler’s behavior, the testing environment, engagement of the preschooler during the task. The single item was rated on a three-point scale where 1 indicating *I have serious concerns about this data*, 2 indicating *I feel ok about this data* and 3 indicating *I feel good about this data*.

## Results

### Feasibility of the EF Touch

Following the previous studies [15, 24] feasibility of EF touch with Pakistani preschoolers was carried out using descriptive analysis. The feasibility report included the *rate of completion* which was defined as the proportion of preschoolers who completed the task; where completion was defined as passing the training items by the preschoolers; *length of administration* which is the time associated with the completion of a task (See Table 1), excluding the breaks or pauses which the preschoolers took between the task; assessor subjective impression of the *quality of data*.

**Table 1:** Frequency and percentage of Executive function task metrics (N = 120).

	Task Summary			Item Summary			
	Tasks attempted (% completion)		% task items completed	Task length(min)	Task item Correct	Floor	Ceiling
Task	*F*	*%*	*M*	*M*	*%*	*%*	*%*
Arrows	116	96.7	1.00	3.5	72.6	0	7.5
Silly Sounds Game	112	93.3	1.00	1.91	71.06	0	5.0
Houses	104	86.7	0.98	5.99	65.1	0	1.7
Something’s the same	106	88.3	1.00	4.24	73.8	0	0.8
Pig	107	89.2	1.00	3.44	91.8	0	21.7
Pick the Picture	117	97.5	0.97	4.6	74.4	0	0
Farmer	95	79.2	0.99	3.49	52.27	0	0.8
Bubbles	120	100	1.00	0.055	98.6	0	85

Note. f = frequency of children who were presented a task and who successfully completed the training items of a task; Floor and Ceiling refer to the proportion of children who completed 0 or 100% of test items correctly.

**Fig 1:**
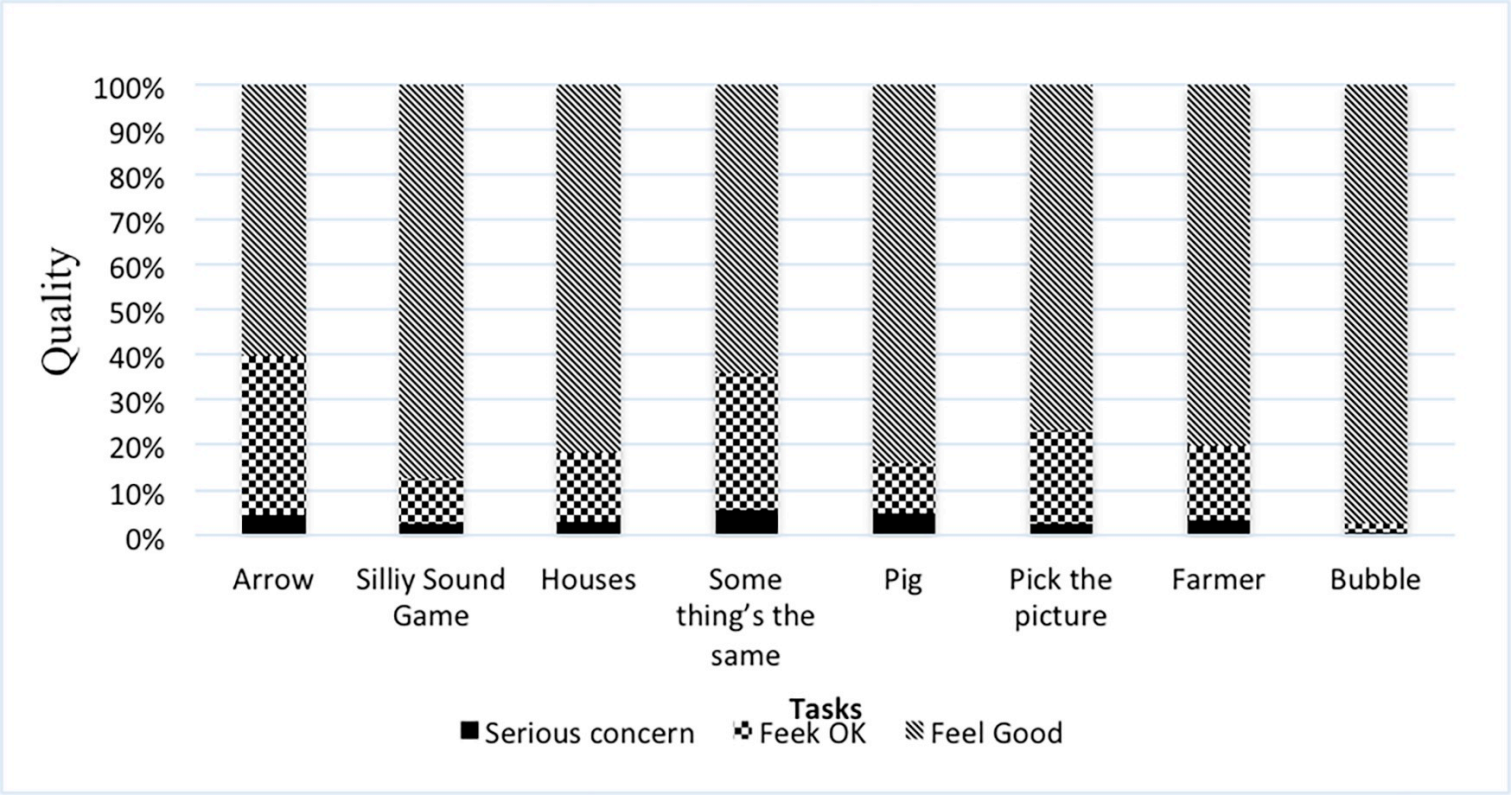
Executive Function task quality ratings (*N* = 120).

Table 1 depicts that between 79.2% and 100% of the preschoolers completed each executive function task. Further, Preschoolers who passed the training items for a given task typically completed the task items too (*M* = 0.97 to *M* = 1.00). Further, the task took an average of 3 to 5 minutes to complete. Table 1 also presents item summary; it reveals that preschoolers completed 52.27% to 98.6% of the test items correctly. The floor and ceiling effect is also evident from the table, for each task 0% preschoolers’ floor effect for the present sample. Ceiling effect are evident, for inhibitory control tasks especially Arrow (7.5%) and for Pig (21.7%) tasks were higher. For working memory tasks Pick the Picture, there was no ceiling effect. Further, Fig 1, shows the subjective evaluation of data quality in each task, on average more than 80% of all administrations were considered to be of good quality or in an acceptable range.

### Dimensionality of EF touch

Each Task of EF touch was developed to be unidimensional. To evaluate the dimensionality of the battery, CFA analysis was carried out. The CFA model of each task corresponding to the different tasks as well as obtained factor loadings of each task was in the respective dimension. All the factor loadings for EF tasks range from λ = .34 to λ = .73. All items have factor loadings in the acceptable range i.e. more than .30 as per criteria (Field, 2009). Further, Table 2 represents the model fit indices for EF Tasks. It shows that model fit χ^2^(df) = 36.18(20) is with values of CFI = .89, IFI = .90 and RMSEA = .08. The value of RMSEA was high so to get a better fit, error covariance was added on basis of content overlapping. The value of RMSEA lowered to .05 which is considered as a good fit.

**Table 2:** Fit indices of confirmatory factor analysis for Executive Function Touch battery.

	χ^2^ (*df*)	NFI	IFI	TLI	CFI	RMSEA	Δχ^2^(*df*)
Model 1	36.18(20)	.81	.90	.86	.89	.08	
Model 2	24.36(18)	.87	.96	.94	.96	.05	11.72(2)

Model 1 = Default model of CFA

Model 2 = M1 after adding error co-variances

### Differential item functioning & item parameter estimation using item response theory

Recognizing the unidimensionality of the EF task battery, the Binary item model was implemented using a two-parameter logistic model (2PLM) was utilized using Item Response Theory (IRT). EF Touch involves the responses of the preschoolers; the responses are dichotomous in nature, either correct or incorrect. The 2PLM has difficulty and discrimination parameters. An item with a large discrimination value has a high correlation between the out latent construct i.e. executive function and the probability of answering the item correctly. The larger the discriminating parameter, the better it can distinguish between low and high levels of executive function ability. Theoretically, the discriminating parameter ranges from 0 to infinity. Using model parameterization used for the present study, values greater than 1 are desirable here. The difficulty parameter, the item location or item intercept, is interpreted as a z metric, it represents the location of an item on the task of the latent trait of executive function of the preschooler. Negative values indicate easy items, values near ’0’ indicative of average difficulty, and positive values indicate difficult items. Further, Differential Item Functioning (DIF) was assessed for the item of each task of the battery for preschoolers who were residing in low (*n* = 66) and middle (*n* = 54) income households. The low-income status was defined by the Pakistan business recorder in terms of the composite index and it falls within the 50 thousand rupees range in low income and more than falls in the middle-income group. DIF was assessed to see that whether items of each task that are intended to investigate the latent ability of executive function were favoring the one-income household groups over the other using Logistic regression DIF.

Table 3 shows that no significant DIF was found for any of the items on each task for the Pakistani sample. Further, item discrimination parameters explaining the preschoolers’ performance on each task have variability in the strength of the relationship between individual items and underlying ability, i.e. preschooler’s performance on each task item was moderate to strongly discriminating. Moreover, the table shows the range of difficulty levels of each task of the battery for the present sample.

**Table 3:** Differential item functioning & item parameter estimation of each EF Task.

Task	Differential Item Functioning	Item Discrimination		Item Difficulty	
		Index	Interpretation	Index	Interpretation
Arrows	Non-significant	.25 to 3.50	Substantial	-3.36 to -.14	Easy
Silly Sounds Game	Non-significant	.56 to 1.82	Moderately To Strongly	-2.47 to -.56	Easy
Houses	Non-significant	-.27 to 1.97	Moderately To Strongly	-4.98 to 5.05	Full Range of Difficulty
Something’s the same	Non-significant	-.56 to 2.70	Moderately To Strongly	-53.18 to 5.56	Full Range of Difficulty
Pig	Non-significant	.57 to 6.49	Substantial	-3.37 to -1.27	Easy
Pick the Picture	Non-significant	-.55 to 2.29	Substantial	-69.18 to 149.84	Full Range of Difficulty
Farmer	Non-significant	.32 to 2.47	Moderately To Strongly	-4.34 to 1.07	Full Range of Difficulty

DIF = Differential Item Functioning

### Test information curves

**Fig 2:**
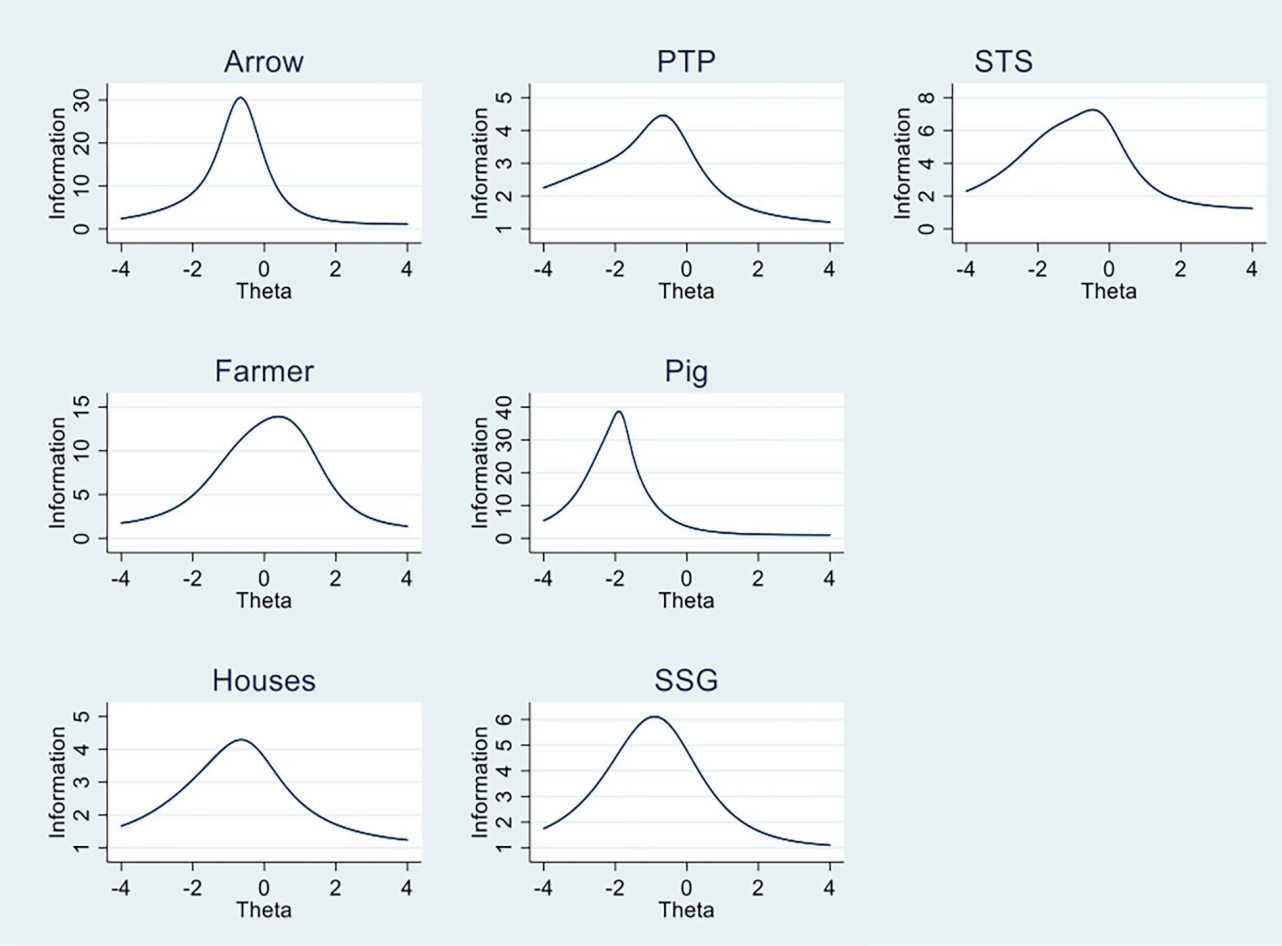
Test information curves of arrow, Silly Sound game (SSG), Houses, Something’s the same (STS), Pig, Pick the picture (PTP), and farmer task +-4 SD around the mean level of executive function ability.

IRT provides the ability to assess how efficiently a test performs over the range of its latent construct it was designed to measure; it can be evaluated in terms of *test information*. It provides reliability or accuracy of a single item or complete scale over the range of its latent ability (theta). For a set of items, the height of the test information curve at any given level of theta shows the strength of items (the slopes) that make the test. The higher test information curves represent the smaller standard error of measurement. Along the dimension of theta, the peak of the test information curve represents by the difficulty parameters of the items that make up the test [23]. Each item for executive function tasks have a different number of items, therefore Test information curves of each task are represented separately (see Fig 2).

## Discussion

The current study is a contribution to limited researches conducted so far on early EF of the children residing in LMIC, using a performance-based measure. This study not only assessed the feasibility of using computer-based assessment for Pakistani preschoolers which were comprehensively used in the high-income country like the United States, but also it reviewed the task, translated the task into the national language Urdu. Dimensionality of the entire battery was assessed. Further, IRT was used for preliminary testing and evaluation of psychometric properties of each task of the battery on a small sample of Pakistani preschoolers.

The objective of translation and content review was to allow precision in the research process among preschoolers from Pakistani sub societies. Although the medium of instruction in several public and private schools is English, most of the preschoolers were not able to understand the English verbal instructions that the assessor used to guide the preschooler to perform the tasks of the battery. The research demonstrated that the translated instructions were understandable and directed the preschoolers effectively. During the review of the tasks, the local professionals and experts (two child psychologists and three social psychologists) recommended minor suggestions, which were not incorporated due to copyrights. For example, they recommended replacing the animal *pig* with some other animal since the pig is not a very common animal in Pakistan. Further, people even dislike listening to or speaking the word ’pig’ due to their religious ideas and cultural values. During the pretest, it was found that this minor concern had not affected the response of the preschoolers. Hence, During the pretest, it was found that this minor concern had not affected the response of the preschoolers. Hence, the quality of assessment procedure was not compromised. Before the recruitment of each preschooler, it was made sure that the preschoolers were familiar with the phenomenon of a touch screen. For each task, the training items ensured that the task was comprehended by the preschoolers. The feasibility metric of the present study demonstrated that most of the preschoolers completed each task of the battery, with minimum, for working memory *Farmer* task; only 79.2% of the preschoolers passed the training items. These results are different from the previous study [15] conducted in LMIC using the same battery, they found the *Arrow* task as most difficult. These findings may suggest that either the criteria for success of training items or the instructions to comprehend the task need revision for Pakistani sample. It is also recommended to test it on larger sample for clearer picture. Further, the performance of preschoolers on each EF task vary and ranged from 52.27% - 91.8%. For the present study, the Pakistani preschoolers showed low performance scores on working memory tasks *Farmer* and *Houses*. One of the possible reasons for low performance might be the environmental condition in LMIC [26].

The quality of rating that was evaluated by the assessor after the completion of each task also supported the feasibility of battery. The results showed that for most of the tasks the assessor was satisfied and felt confident with the data quality. Additionally, it demonstrated that most of the preschoolers understood the task well; they were fully engaged and involved during the task.

To evaluate the dimensionality of the entire battery, CFA analysis was carried out. The results demonstrated the unidimensionality of the EF battery for the current sample. It added to previous researches [15, 27, 28, e.g. 29, 30] that examined the dimensionality of early EF in children and found it to be unidimensional. According to Jurado and Rosselli [31], executive function skills may develop from a unitary factor very early in life but differentiate during childhood when multiple, separable control processes evolve.

Differential Item Functioning (*DIF*) analyses were carried out concerning preschoolers residing in low and middle-income households. Within IRT, DIF assesses that whether items of each task that are intended to investigate the latent ability of executive function were favoring the one income household groups. The results demonstrated that no DIF was revealed for any of the tasks of the battery for the Pakistani sample; suggesting that differences in performance between the two groups may not be associated with the biases in test construction and this test works equally well for both income groups. These findings are consistent with the previous work [21, 23] conducted with children residing in low and middle-income families.

Further, using the standardized procedure of IRT; item characteristic was evaluated for each task of the battery. The item parameters identified the items that worked well or poorly including to what extent they considered to be difficult or easy for each task. TIC’s showed the efficiency of the battery over the range of its latent EF ability. Fig 2 represents the range of information for EF for the present sample reflecting their true level of ability peak between 2 Standard Deviation (SD) below and high the population mean of EF ability. The *Arrow* and the *Pig* tasks provide more information with high curves but for the Pig task, the peak is below the mean around 2SD of EF ability. The results of each task of the battery provide reliable scores for the present sample, and their true ability ranges from relatively moderate average level.

Previous studies [16, 32] have highlighted culturally specific issues of using the cognitive assessment tools in LMIC that are developed in high-income countries. One of the major concerns is the utilization of specific testing material and strategies, as mentioned earlier, the utilization of a touch screen. Before data collection the preschoolers were tested and it was inquired if the preschooler are familiar with the concept of touch screen or not, further, EF touch was introduced to them as a fun activity and they were asked to enjoy it. The researcher made sure that preschooler don’t get an impression that they are being tested at any point during the assessment, even if they were not able to pass the training items. The preschoolers were praised throughout the assessment procedure. Another apprehension is the one-to-one relationship during the assessment procedure; though for Pakistani samples, such kinds of assessment are not a routine activity. However, the study preschoolers were comfortable and were not dependent on social collaboration for the task involved in the EF touch, as expected and suggested in a social group-oriented society [33]. The present research addressed and accommodated the major issues that are considered to threaten the use of cognitive assessment tools developed in high-income countries.

### Limitations of the study

The present study demonstrated an important contribution to conduct a preliminary study to assess early EF skills using the performance-based measure in preschoolers from LMIC like Pakistan. Nevertheless, the current results should be considered in the context of few limitations, which need to be considered for future studies. Though it was a preliminary study, however, the sample size was small and was collected through convenient sampling. While the sample represented diversity in terms of selection of schools from public i.e. government-funded and private schools but still it is recommended to withdraw a large sample from a more diverse population that covers not only urban but also rural sample in future for the bigger picture. Moreover, it is recommended to use modern testing theories on a larger sample however, smaller sample size is sufficient for preliminary analysis [34, 35]. Further, the data was collected in the national language Urdu only, though some of the students were proficient in English as well as Urdu, they should have given a chance in the language of their choice. Moreover, the Android version or smaller gadget is recommended over the current Windows version owing to its technical requirements.

## Conclusions

Despite these limitations, the results are promising. Initially, during the pretesting, it was apprehended that the preschoolers from the low-income household may not be comfortable or comprehend the use of touch screen or it may affect the performance but during the data collection it was observed that the preschoolers were fully absorbed and enjoyed the tasks and it acquired little effort to engage the preschoolers. The merit of the current performance-based measure is characterized by no rigorous training for the assessor; the programmed based decision of whether the preschooler passed the training item, ease of breaks during the made the data collection process smooth. Further, the IRT analysis used in the present study revealed the reliability of each task of the battery and the preschoolers’ latent ability to perform each task. In conclusion, findings from the present study demonstrated the effectiveness and feasibility of the EF Touch battery to assess early EF skills in preschoolers in Pakistan and may provide an avenue for future researches in LMIC by providing a culturally feasible, reliable, and valid instrument. It will also give direction to the new researchers to adapt the various performance-based instrument to a more friendly computerized version and will assist to comprehend the variation caused by environmental input of developing countries on higher-order cognitive functioning in early childhood. Further, the present research can facilitate policymakers to highlight issues related to pedagogy and early education.

## Supporting information

S1 File For EF touch battery details. (PDF)

S2 File Dataset file of EF touch battery. (SAV)
