# Supplementary material for: Executive Function Touch battery: Translation and preliminary measure validation for Pakistani preschoolers
Source: PLoS One. 2022 Sep 15;17(9):e0274431. doi: 10.1371/journal.pone.0274431 (PMC9477268; doi:10.1371/journal.pone.0274431)
Supplement: S1 File — (PDF) [file pone.0274431.s001.pdf]

Introduction

How to use this manual

This manual is divided into seven chapters and is intended for researchers interested in using our EF battery, as well as for assessors who will be administering it. The individual chapters can be used as stand-alone documents. However, for a complete understanding of the EF battery we advise reading the entire manual. Within this manual you will find a detailed description of the individual tasks and how they were developed through our extensive pilot work and testing. The manual also provides a detailed description of the psychometric properties of the battery and how this provides insight into how the battery should be administered. Finally, in this manual, you will find comprehensive information on how to administer the battery and interpret the scores.

Chapters one, two and three will be most useful for researchers and students interested in a comprehensive overview of how the individual tasks were selected, modified, and conceived of as a single indicator of EF. Chapter two specifically provides information on the psychometric properties of the battery and how it was validated using a large representative sample. This chapter may also be informative to those who are interested in the quantitative work that has been conducted with the battery. Chapter three explains the process of transitioning into a computerized assessment and provides some information on the psychometric properties of the battery in this format.

Chapters four, five and six will be most useful for assessors, who will be administering the battery. Chapter four provides the most detailed information on the individual tasks and how they are administered. This chapter was written to promote assessor comprehension and to

answer questions that commonly occur during administration. Chapter four will also be important for researchers who are planning on selecting certain tasks, from the complete battery, based upon content interests or time restrictions. Chapter five provides specific details and tips for administering the battery. This chapter only provides brief descriptions of the individual tasks, for more detailed descriptions please read chapter four. Chapter six provides a technical overview of how to set up the equipment and should be read by all assessors before administering the battery.

Chapter seven will be utilized be anyone responsible for managing data or conducting analyses with data from the EF Touch battery. This chapter defines variable names and contains recommendations on how to use specific variables for scoring proposes.

Chapter One

History of Executive Function Tasks and Task Overview

Executive function (EF) is an “umbrella term” that refers to a wide range of cognitive abilities involved in the control and coordination of information in the service of goal-directed actions (Fuster, 1997; Miller & Cohen, 2001). As such, EF can be defined as a supervisory system that is important for planning, reasoning ability, and the integration of thought and action (Shallice & Burgess, 1996). At a more fine grained level, however, EF, as studied in the cognitive development literature, has come to refer to specific interrelated information processing abilities that enable the resolution of conflicting information; namely, working memory, defined as the holding in mind and updating of information while performing some operation on it; inhibitory control, defined as the inhibition of prepotent or automatized responding when engaged in task completion; and mental flexibility, defined as the ability to shift attentional focus or what is referred to as cognitive set among distinct but related dimensions or aspects of a given task (Garon, Bryson, & Smith, 2008; Zelazo & Müeller, 2002).

Here we summarize our ongoing efforts to develop and rigorously evaluate a battery of executive function tasks for use across the early childhood period (i.e., 3-5 years of age). When we began working in this area approximately 7 years ago, there were relatively few task batteries available that represented the tripartite organization of EF (inhibitory control [IC], working memory [WM], attention shifting [AS]), that had undergone rigorous psychometric evaluations—including use with children from disadvantaged settings that yielded scalable scores to facilitate estimates of within person changes in ability, and that were amenable for use by lay interviewers (without expertise in EF) in large scale, field-based settings.

Under the auspices of NICHD funding (R01 HD51502), we sought to develop a test battery that addressed these limitations. Specifically, we were interested in developing tasks that represented a broad range of ability level and that yielded individual differences in EF ability levels between 3-5 years of age. Moreover, we wanted tasks that were highly portable, that presented tasks in a uniform format, and that included highly scripted instructions, making them amenable for use by lay data collectors who had limited experience in standardized testing and no content knowledge of EF. In addition, we constructed tasks such that one person administered the tasks while a second person recorded child responses, in order to reduce the cognitive demand for data collectors and improve data quality. Finally, we were interested in tasks that were both appropriate for young children and that were ostensibly designed to measure the distinct components of EF (understanding that there are no completely "pure" measures of WM, IC, or AS) that have been critical for testing fundamental questions about the organization of EF in early childhood.

Our work proceeded in three stages. The first stage involved an iterative process of task development and pilot testing and concluded by administering the first complete task battery in a cross sectional sample of children between the ages of 3 and 5 years. The second stage involved introducing the task battery into a population-based longitudinal sample of 1,292 children and families followed from birth (i.e., the Family Life Project; P01 HD39667). The third stage involved a new round of task revisions that were intended to facilitate the migration of the battery from a flip book to computer-based administration format.

Initial Task Development

We initially set out to identify *extant* tasks that had been used with preschool age children that putatively measured three aspects of EF—inhibitory control (IC), working memory (WM), and attention shifting (AS)—and that could be adapted for use with very young children who resided in low income households. The focus on young and disadvantaged children was to facilitate our second stage of psychometric work, which involved direct assessments of EF in 3-year old children who were part of the Family Life Project, a NICHD-funded population based cohort that over-sampled low income and, in NC, African American families.

The initial set of tasks was developed as a series of "card games" that were intended to provide a uniform testing format. Nine potential tasks were trimmed to six following our experiences with pilot testing (some tasks were difficult for the youngest children to understand). Pilot testing also revealed that a "card game" format was not ideal, as cards required sorting after each administration and the likelihood of sorting-related errors was large. We subsequently migrated the tasks into a series of bound "flip books," which eliminated sorting problems and facilitated the scripting of instructions for administrators (the opened flip book sat between the experimenter and the child; one page presented a stimuli to the child while the other page provided scripted instructions that were read to the child by the experimenter or that otherwise prompted the experimenter as to what to do). We worked with a commercial artist to develop professional looking stimuli, a graphic designer and printer to construct bound flip books, and a computer programmer who developed software to record child responses into laptops.

Using the flip book format, we engaged in an extended period of iterative pilot testing and task modification with a total of 120 children. During this period, no formal data were collected. Rather, two data collector teams independently tested children to gain impressions of how tasks worked and what modifications might be useful. Conference calls involving data

collectors and investigators led to recommended changes, implemented by a graphic designer and subsequently pilot tested again. Major emphases were developing language that described task objectives in ways that were understandable to young children and structuring tasks in ways that made minimal language demands on children in order to respond. A synopsis of the final set of tasks retained from this initial task development period follows.

Task Descriptions

Two tasks were developed that putatively measured working memory. The Working Memory Span (Houses) task was based upon principles described by Engle, Kane and collaborators (e.g., Kane & Engle, 2003) in which the object is to hold multiple representations in mind simultaneously and to selectively respond to only one. In this task, children are presented with a line drawing of an animal with a colored dot above it, both located within the outline of a house. The examiner asks the child to name the animal and then to name the color of the dot. The examiner then turns to a page which only shows the outline of the house from the previous page, and asks the child which animal was/lived in the house. The task requires children to perform the operation of naming and holding in mind two pieces of information simultaneously and to activate the animal name while overcoming interference occurring from having named the color. The number of houses on each page scales difficulty level. The Pick the Picture (PtP) task is a self-ordered pointing task (Cragg & Nation, 2007; Petrides & Milner, 1982). Children are presented with a set of identical pictures that appear on a series of consecutive pages. On each page, they are instructed to pick a new picture that had not been previously selected so that all of the pictures "get a turn." The arrangement of pictures within each set is randomly changed across trials so that spatial location is not informative. The number of pictures contained in each set scales difficulty level.

Three tasks were developed that putatively measured inhibitory control. The Silly Sounds Stroop (SSG) task was derived from the Day-Night task developed by Gerstadt, Hong, and Diamond (1994). While showing the children pictures of a cat and dog, the experimenter introduces the idea that, in the Silly Sounds game, dogs make the sounds of cats and vice versa. Children are then presented with pictures of dogs and cats and asked what sound a particular animal makes in the Silly Sounds game (i.e., children are to 'bark' for each cat picture and 'meow' for each dog picture). The second task that putatively measured inhibitory control is the Animal Go/No-Go (Pig) task. This is a standard go no-go task (e.g., Durston et al., 2002) presented in a flipbook format. Children are presented with a large button that makes a clicking noise when depressed. Children are instructed to click their button every time that they see an animal on the flip book page unless that animal is a pig. Each page depicts one of seven possible animals. The task presented varying numbers of go trials prior to each no-go trial. The third task that putatively measured inhibitory control is the Spatial Conflict (SC) task. The SC is a simon task inspired by the work of Gerardi-Caulton (2000). Children receive a response card with a picture of a car on the left side and picture of a boat on the right side. The flip book pages depict either a car or boats. The child is instructed to touch the car on his/her response card when the flip book page shows a car and to touch the boat on the response card when the page shows a boat. Initially, cars and boats are depicted in the center of the flip book page (to teach the task). Across a series of trials, cars and boats are depicted laterally, with cars (boats) always appearing on the left (right) side of the flip book page ("above" the car [boat] on the response card). Eventually test items begin to appear in which cars and boats are depicted contra-laterally (spatial location is no longer informative). In subsequent work, it was determined that the SC task was too easy for 4 and 5 years-olds. Hence, a variation of the task, called the Spatial

Conflict Arrows (Arrows) task was developed. It was identical in structure to SC with the exception that the stimuli were arrows and the response card showed two “buttons” (black circles). Children were to touch the left (right) button when the arrow on the flip book paged pointed to the left (right) side of the page. Similar to SC, initially all left (right) pointing arrows appeared above the left (right) button; however, during test trials left (right) pointing arrows appeared above right (left) button.

One task was developed that putatively measured attention shifting. The Something’s the Same (STS) task was a simplified version of Jacques and Zelazo’s (2001) flexible item selection task. In our variation, children are shown a page containing two pictures that are similar along one dimension (content, color, or size). The experimenter explicitly states the dimension of similarity. The next page presents the same two pictures, plus a new third picture. The third picture is similar to one of the first two pictures along a dimension that is different from that of the similarity of the first two pictures (e.g., if the first two pictures were similar in shape, the third card would be similar to one of the first two along the dimension of color or size). Children are asked to choose one of the two original pictures that is the same as the new picture.

Chapter Two

Factor Structure, Measurement Invariance, Criterion Validity & Age Differences EF

After the previously described iterative pilot testing, data were collected from a convenience sample (*N* = 229 children) in NC and PA. Children ranged from 3.0 to 5.8 years of age and were recruited to ensure adequate cross-age variation. This sample was used to conduct confirmatory factor analyses (CFA) with the EF battery. A primary analytic objective of developing this battery was to test its dimensionality, to test whether it exhibited equivalent measurement properties for distinct subsets of youth, to examine the criterion validity, and to evaluate whether performance exhibited the expected improvements as a function of chronological age.

First, our EF tasks were best represented by a single latent factor, $\chi^2_{(9)} = 4.0$, $p = .91$, CFI = 1.0, RMSEA = 0.00. With one exception, the EF latent variable explained approximately one-third of the variance of each task (Spatial Conflict: $R^2 = .07$; Go No-Go (Pig): $R^2 = .29$; Working Memory Span (Houses): $R^2 = .34$; Silly Sounds Stroop (SSG): $R^2 = .40$; Item Selection (STS): $R^2 = .41$; Self Ordered Pointing (PTP): $R^2 = .47$). Nearly two-thirds of the observed variation in each task was a combination of measurement error and systematic variation unique to that task. This provided evidence in favor of pooling information across tasks in order to establish a reliable index of latent EF ability. Moreover, the excellent fit of the 1-factor model argued against the relevance of the tripartite organization of EF in early childhood.

Second, a series of multiple groups CFA models were estimated in order to test the measurement invariance of the 1-factor model separately by child sex (male vs. female) and age group (3-3.99 vs. 4-5 years), parental education (less than vs. 4+ year degree), and household

income (median split of $50,000/household). That is, we tested whether tasks were equally good indicators of the construct of EF across subgroups of children. At least partial measurement invariance was established for all comparisons (i.e., at least a subset of the tasks could take on equivalent measurement properties across all subgroups of children). This provided evidence that the task battery "worked", in a psychometric sense, equivalently for a wide range of children. Measurement invariance facilitated our ability to make meaningful cross-group comparisons. Comparisons of latent means indicated that girls outperformed boys (Cohen $d = .75$), older children outperformed younger children (Cohen $d = 1.3$), children whose parents had a bachelor's degree or higher outperformed children whose parents did not (Cohen $d = .54$), and children from higher income households outperformed children from lower income households (Cohen d = 0.44) on the EF battery (all *p*s < .01).

Third, in the total sample, the EF latent variable was negatively correlated with a latent variable of parent-reported attention deficit hyperactivity disorder (ADHD) symptomatology ($\varphi = -.45$, $p < .0001$) and positively correlated with a direct screening assessment (i.e., WPPSI subtests) of children's IQ ($\varphi = .77$, $p < .0001$). These results were in the expected direction and provided initial evidence supporting the criterion validity of the battery. Fourth, EF factor scores were positively correlated with chronological age ($r = .55$, $p < .0001$) and exhibited linear change from age 3-6 years. An inspection of box and whisker plots of EF factor scores plotted by age group demonstrated that although children's performance on the EF battery showed the expected linear changes with increasing age, there were substantial individual differences in ability level within any given age (see Figure 1). The ability of the EF task battery to preserve individual differences within age group makes it an ideal measure for measuring longitudinal change.

In sum, at the conclusion of the first stage of our measurement development work, we had developed a set of tasks that had undergone extensive pilot testing and revision, which were presented in a uniform and easily portable format, and which could be easily administered by data collectors with no expertise in EF. Analyses indicated that children's performance on six tasks was optimally represented by a single latent factor and that EF tasks worked in an equivalent way for children of different sexes, ages, and family backgrounds. Group differences in latent EF ability were evident for females vs. males, older vs. younger children, and children from more (parental education, household income) vs. less advantaged homes. Criterion validity was established by demonstrating that children's performance on the battery correlated with parent-reported ADHD behaviors and their performance on two screening indicators of IQ. Finally, although there was evidence of developmental improvements in performance on the battery, individual differences in ability were evident for children of the same age(s).

Psychometric Properties of the EF Battery in a Large Scale Evaluation

With the successful completion of measure development activities in the pilot testing, the EF task battery was administered at the age 3, 4, and 5-year assessments of the Family Life Project (FLP). The FLP is a NICHD-funded program project that was designed to study young children and their families who lived in two of the four major geographical areas of the United States with high poverty rates. Specifically, three counties in Eastern North Carolina and three counties in Central Pennsylvania were selected to be indicative of the Black South and Appalachia, respectively. The FLP adopted a developmental epidemiological design in which sampling procedures were employed to recruit a representative sample of 1,292 children whose families resided in one of the six counties at the time of the child's birth. Low-income families in both states and African American families in NC were over-sampled (African American

families were not over-sampled in PA because the target communities were at least 95% non-African American). Interested readers are referred to a monograph-length description of study recruitment strategies and detailed descriptions of participating families and their communities (Vernon-Feagans, Cox, and the Family Life Project Key Investigators, 2011). Here, we summarize the knowledge that was gained, specifically as it related to our EF task development efforts, from embedding the EF battery in the FLP study.

The inclusion of the EF task battery at the age 3 year assessment of the FLP sample provided the first sufficiently large sample to begin evaluating the psychometric properties of each task individually, as well as their contribution to the overall battery. Three key results emerged from this study (see Willoughby, Blair, Wirth, Greenberg, & Investigators, 2010). First, 91% of children who participated in the age 3-year home visit successfully completed one or more EF tasks (median = 4 of 5 tasks; note that the PTP task was not administered at the age 3 assessment). Compared to children who completed one or more EF tasks, children who were unable to complete any tasks were more likely to be male (77% vs. 47%, $p < .0001$), to have a primary caregiver who was unmarried (57% vs. 42%, $p = .005$), and to differ on their estimated full scale IQ score using two subtests from the WPPSI ($M = 75.9$ vs. $M = 95.1$, $p < .0001$). The WPPSI scores, in particular, indicate that children who were unable to complete any EF tasks were developmentally delayed. Second, a CFA indicated that a 1-factor model fit the task scores well ($\chi^2_{(5)} = 3.5$, $p = .62$, CFI = 1.0, RMSEA = 0.00). Although a 2-factor model fit the task scores well too ($\chi^2_{(4)} = 2.4$, $p = .66$, CFI = 1.0, RMSEA = 0.00), the 2-factor model did not provide a statistically significant improvement in model fit relative to the 1-factor model, ($\chi^2_{(1)} = 1.1$, $p = .30$). Hence, consistent with other relevant studies and the results from data collected in pilot testing, EF abilities were again best conceptualized as unidimensional (undifferentiated) in

preschoolers. Third, a final set of CFA models were estimated to establish the criterion validity of the EF task battery. One latent variable represented children's performance on the EF task battery (each task was an indicator of underlying EF ability). The other two latent variables represented criterion measures: multiple informant (parent, day care provider, research assistant) ratings of ADHD behaviors and estimated intellectual functioning (based on performance on Block Design and Receptive Vocabulary subtests of the WPPSI). This 3-factor model fit the observed data well ($\chi^2_{(32)} = 36.0$, $p = .29$, CFI = 1.0, RMSEA = 0.01). The EF latent variable was strongly negatively correlated with ADHD ($\varphi = -.71$, $p < .001$) and strongly positively correlated with IQ ($\varphi = .94$, $p < .001$). These results replicated those obtained in Stage 1. Although the correlation between EF and IQ approached unity, it was in line with the range of effects that were observed in the adult literature (Kane, Hambrick, & Conway, 2005).

In addition to replicating tests regarding the dimensionality and criterion validity of the EF *battery*, the large sample size of the FLP provided the first opportunity to evaluate the psychometric properties of *individual tasks*. We relied extensively on Item Response Theory (IRT) methods for psychometric evaluation of individual EF tasks. IRT methods had three advantages that were directly relevant to our work. First, IRT methods provide a principled approach for testing differential item functioning (DIF). DIF is a formal approach for evaluating whether *individual items* on each EF task work equally well for subgroups of participants. Ruling out DIF ensured that any observed group differences in ability were not an artifact of differential measurement operations. Second, IRT methods provided an explicit strategy for generating task scores that took into account the fact that test items varied with respect to their difficulty level and discrimination properties. Failing to take into account differences in how each item behaves results in under- or over-weighting particular items. Incorrectly weighting items can lead to scale

scores that are biased and thereby less accurate when comparing individuals (or groups) within or across time (Edwards & Wirth, 2009; Wirth & Edwards, 2007). A third advantage of IRT methods was the ability to compute test information curves, which characterize variations in the precision of measurement of each task as a function of a child's true ability level. Test information curves consistently indicated that *all* of the tasks in our battery did a relatively better job of measuring lower than higher levels of true EF ability. These results informed subsequent efforts at task revisions (see below). We used data from the age 4-year assessment to provide an extended introduction to the merits of IRT methods for purposes of task evaluation (Willoughby, Wirth, & Blair, 2011).

A test-retest study was embedded in the FLP age 4-year assessment (Willoughby & Blair, 2011). Over a 6-week period, every family who completed a home visit (n = 145) was invited to complete a follow-up (retest) visit 2-4 weeks later. The follow-up visit consisted of the re-administration of EF tasks by the same research assistant who administered them at the first visit. Of the 145 families who were invited to participate, 141 accepted (97%). The mean (median) number of days that passed between the initial and retest visits was 18 (16) days, with a range of 12-42 days. One child whose family agreed to participate in the retest visit was unable to complete any tasks, yielding 140 retest observations. Retest correlations of individual task scores were moderate (mean r = .60; range = .52 - .66). Although similar in magnitude to those observed in other retest studies of EF in preschool, school-aged, and adult samples, we were discouraged by these low values given the short retest interval (i.e., only 1/3 of the observed variation in performance on any given EF task was shared across a two-week period). However, in contrast to individual tasks, confirmatory factor analyses demonstrated very high retest reliability (φ = .95) of the task battery. This pattern of results was identical to those of an earlier

study that demonstrated this approach with older adults (Ettenhofer, Hambrick, & Abeles, 2006). We concluded that children's performance on any individual task consisted of a combination of measurement error, systematic variance that was task specific and systematic variance that represented general EF ability. Through the administration of multiple tasks, we were able to extract that variation that was common across EF tasks. The large retest correlations between latent variables demonstrated a high degree of stability of the variation in ability that was shared across tasks. Perhaps more than any other manuscript that we published, this study underscored the critical importance of administering multiple tasks and defining EF ability using latent variable models that were defined by that variation that was common (shared) across tasks.

The inclusion of the EF task battery at the FLP age 5-year assessment yielded three major findings (Willoughby, Blair, Wirth, Greenberg, & Investigators, 2012). First, 99% (N = 1,036) of the children who participated in this visit were able to complete one or more of the tasks (median = 6 of 6). This represented an 8% increase in the completion rate from the 3-year assessment. As before, those children unable to complete any tasks tended to exhibit a variety of physical and developmental delays that prohibited their participation in any direct child testing. Given the inclusive nature of the FLP sampling scheme (over-sample low income families, no exclusionary criteria except intention to move and speaking English as a primary language), the 99% completion rate provided direct support for the use of this battery with children from diverse backgrounds. Second, also consistent with results from the age 3-year assessment, children's performance on EF tasks continued to be optimally characterized by a single factor, $\chi^2_{(9)} = 6.3$, $p = .71$, CFI = 1.0, RMSEA = 0.00. This indicated that the differentiation of EF ability into more fine grained dimensions likely occurs after the start of formal schooling (see Shin et al., in press, for supporting evidence). Third, a series of CFA models, based on N = 1,058

children who participated in either the age 5 year and/or pre-kindergarten (i.e., a visit that occurred prior to the start of kindergarten) visits, were used to test whether children's performance on the EF battery was significantly related to performance on achievement tests. A 2-factor model (one each for EF and academic achievement) fit the data well, $\chi^2_{(43)} = 135.1$, $p < .0001$, CFI =.96, RMSEA = 0.05. The latent variables representing EF and academic achievement were strongly and positively correlated ($\varphi = .70$, $p < .001$). Moreover, follow up analyses indicated that the EF latent variable was moderately to strongly correlated with individual achievement scores as well (φ ECLS-K Math= .62, φ WJ Applied Problems = .63, φ WJ Quantitative Concepts = .62, φ WJ Letter-Word = .39, and φ TOPEL Phonological = .56, all *p*s < .001). These results validated the EF battery as an indicator of academic school readiness.

By embedding the EF battery in the FLP, we were able to test the longitudinal measurement properties of the EF battery, as well as characterize the degree of developmental change in children's performance from 3-5 years of age. To be clear, whereas previous results tested the measurement equivalence of items (tasks) across defined subgroups of youth (e.g., gender, family poverty level), here we tested whether the measurement parameters for items on a task and tasks on the battery could take on identical values across time without degrading model fit. Establishing longitudinal invariance of items (tasks) was a necessary precursor to modeling developmental changes in performance across time (failure to establish longitudinal invariance can result in scores that are not on the same metric). Three results emerged from this work (Willoughby, Wirth, Blair, & Investigators, in press). First, all six individual EF tasks exhibited strong measurement invariance across time. That is, item thresholds and factor loadings could take on identical values at 3, 4, and 5-year assessments without degrading model fit. Moreover, the EF battery, defined by a latent variable with the six individual tasks as indicators, exhibited

partial strong invariance over time. Whereas some tasks were differentially strong indicators of EF ability at different ages, two (STS, PTP) had invariant properties that facilitated the creation of a scalable battery score (note that the magnitude of differences in non-invariant tasks was modest; these differences were detected due to excessive statistical power associated with our large sample size). Third, given our previous emphasis on the importance of aggregating children's performance across tasks in order to obtain a reliable indicator of true EF ability, we estimated changes in children's performance on a battery score (i.e., a latent variable estimate of ability at each age) using second-order latent growth curve models. Results indicated substantial age-related improvements in latent EF ability across time (approximately a one-tenth of a standard deviation improvement in ability with each passing month). Moreover, the rate of growth was faster between 3-4 years than it was between 4-5 years (60% of total change between the age 3-5 year assessments occurred between the age 3-4 year assessments). Ongoing analyses are focused on the best ways to represent changes in EF ability across time.

In summary, we replicated and extended the empirical results from pilot testing by demonstrating that children's performance on EF tasks was best represented by a single latent factor at ages 3, 4, and 5 years, and that individual differences in EF ability were correlated with multi-informant ratings of ADHD symptomatology, as well as direct assessments of children's intelligence and academic achievement. Whereas the retest reliability of individual tasks was only moderate, the retest reliability of the latent variable estimate of true ability level was outstanding. In terms of developmental questions, the items on all six EF tasks exhibited strong longitudinal invariance across time, while two of the six tasks exhibited partial strong longitudinal invariance across time (the remaining four tasks were differentially strong indicators of true EF ability at different ages). Extending the results from pilot testing, which were based on

between group comparisons of children of differing ages, this second stage demonstrated substantial improvements in children’s performance on EF tasks across time.

Chapter Three

Developing a Computerized Assessment

Since its inception, our measurement development work has been primarily focused on the creation of a highly portable, easily administered, and uniformly presented battery that assesses the full domain of EF abilities, that is appropriate for serial use (including longitudinal data collection and progress monitoring), and that has been demonstrated to work with preschool-aged children from a wide range of socio-economic backgrounds. Despite some initial success at these efforts, at the end of the second stage of work, we identified three structural and one empirical limitation of the current battery that precluded its wider scale use. First, the flipbook version of the EF battery required two data collectors for administration and scoring. Although this was essential for our large-scale research, the requirement that direct assessment of EF requires two data collectors is both costly and impractical in many potential settings. Second, the flipbook version of the battery required commercial software for recording child responses into laptops, for executing programs that translate the observed child responses into item-level scores, and, when desired, for converting item-level responses into IRT scores. Many potential end-users would not have the interest or the technical sophistication required to use and adapt existing software programs to meet their specific needs, nor would they want to incur the associated annual software licensing costs. Third, although highly structured in content, the flip book version of the tasks do not afford quality control for subtle aspects of task administration that may have major impacts on the quality of data collected (e.g., the rate of presentation of items; an appreciation for how many attempts should be made to teach the task to the child before it is abandoned). Fourth, the tasks did a poorer job of measuring high (advanced) than low (immature) levels of EF ability. An inherent tension in task development is the creation of tasks

that are accessible for children with low ability levels but that also become sufficiently challenging for children with high ability levels.

Using competitively awarded funds from a two-year ARRA supplement to our aforementioned NICHD R01 study, we developed a prototype of a computerized version of the EF battery that was intended to overcome each of these limitations. The computerized battery eliminated the need for two person administration. By relying on touch screen technology, child responses to EF tasks are “captured” by the touch screen (the inclusion of a touch screen eliminates the data collector whose sole purpose was recording child responses). Second, by creating a stand-alone piece of software that delivers test stimuli, records child responses, and provides item-level scoring, we eliminate the costs and technical complications associated with reliance on third-party software solutions. Third, the use of touch screen technology allows the possibility of including reaction time as a supplement to accuracy information for children’s responses to tasks. This may facilitate improved precision of measurement for children at higher ability levels. In the adult literature, reaction time, not accuracy, is the primary individual difference factor discriminating high from low performers on these tasks, and it is likely that reaction time data will also be an important source of information for child performance on the inhibitory control tasks. Fourth, moving to a computerized interface has expedited task development, modification and piloting efforts (graphic design and printing costs and time are eliminated).

Our initial computerization efforts have resulted in a number of important insights. For example, although we initially hoped to use touch-screen tablet computers in order to mimic the layout of our flip book tasks, this was abandoned after we determined that test stimuli appeared distorted to children when images were viewed from an angle (e.g., when children ‘slouched’ in

their seat during testing). Hence, in our current prototype, we use a stand-alone, upright monitor that sits directly in front of the child, which is connected to a laptop that is used by the interviewer. Through pilot testing, we have determined that capacitive touch screen technology is superior to resistive touch screen technology. Only the former registers even the ‘lightest’ of touches, which are common among young children. Capacitive technology also allows children to use their fingers to touch screens, which is more natural than asking them to use a stylus. Finally, through computerization, we are able to standardize multiple aspects of task delivery (e.g., the inter-stimulus time intervals; linking child performance on training items to determination of whether the task should be administered). In the process of computerization, some tasks were necessarily changed so that children could respond via interaction with the touch screen. For example, in the flip book version of the Silly Sounds Stroop (SSG) task, children were asked to either audibly ‘bark’ or ‘meow’ when they were presented with pictures of cats or dogs, respectively. In the computerized version, the computer presents ‘barking’ or ‘meowing’ sounds for which the child have to touch pictures of cats or dogs, respectively.

Following the original translation of tasks from flip book to computerized formats, we engaged in small scale pilot test of *N* = 39 3-5 year-olds. This identified an initial set of problems with the computer interface (e.g., some test stimuli appeared too quickly; inability to re-administer items following an interruption to testing), as well as task improvements (e.g., incorporate graphics and sounds to keep children engaged in the task; introduce a training task to orient children how to respond to questions through interaction with touch screens). The tasks were further refined and the current computerized battery goes by the name EF Touch. In the next stage of development, we collaborated with the Griffin Early Childhood Center Study (GECC) at the University of Chicago, Department of Behavioral Economics. This work was

facilitated by their funded mandate which states, the GECC operates as a “lab without walls”, including issuing an annual request for proposals which include the opportunity to collect data using the GECC sample. We responded to this request for proposals and proposed embedding a test-retest study of the EF Touch battery into their on-going data collection.

Psychometric Work with the EF Touch Battery

A total of N=220 children were tested at the initial assessment, with *N*=191 of these children being retested approximately 2-weeks later. The age range of children assessed was 3.4 to 5.4 years (M = 4.4, SD = .6). Children who participated in both the initial and retest visits (N = 191) did not differ from those who only participated in the initial visit (*N* = 29) with respect to data collector ratings of attention (M = 2.3 vs. 2.3, *t* [215] = -.12, p = .90) or positive behaviors during testing (M = 2.4 vs. 2.5, *t* [215] = .73, p = .46) at the first visit. Moreover, children who participated in both visits did not differ from those who only participated in the initial visit in terms of the number of tasks attempted (M = 4.8 vs. 4.8, *t* [218] = -.16, p = .87) or completed (M = 4.0 vs. 4.3, t [218] = 1.78, p = .08) at the first visit, nor in their age (M = 4.4 vs. 4.5 years, *t* [218] = 1.03, p = .31) or family poverty level (71% vs. 68% poor, χ^2 [1] = .11, p = .74), where poverty level was defined by family use of TANF, food stamps, Medicaid or WIC.

Aggregate Task Performance

In our previous work, we conceptualized each task as an imperfect indicator of the construct of EF, which led to use of each task as an indicator of the latent variable EF using confirmatory factor analysis. The latent test-retest correlation of the latent variable of EF was .95 (Willoughby & Blair, 2011). A two-factor model (one factor for EF at test and retest assessments) in which factor loadings and residual variances were equated to be equal (to ensure

that latent variables were on the same metric) fit the data well, χ^2 [58] = 77.4, p = .05, CFI = .96, RMSEA (90% CI) = .04 (.01 - .07), test of close fit (RMSEA <= .05) p = .70. All factor loadings, as well as residual and latent variances, were statistically significant, ps < .001. With the exception of STS (p = .08), which was the scaling indicator, the residual correlations for each task were all significant (rs = .25 - .63, ps < .05). This indicated that even after extracting the shared variation across tasks to define the latent variable EF, there continued to be significant task-specific variation. The latent variables of EF were correlated .999 across time (essentially unity). As in previous work, despite this high level of stability, the latent variable of EF explained <= 50% of the observed variation in each task. We interpret these results as indicating that the variation that is shared across EF tasks is highly stable. It likely reflects individual differences in attentional capacity, cognitive ability, and the ability to comply with adult requests.

Chapter Four

Using the Executive Function Touch Battery

This Executive Function (EF) assessment is a measure of working memory, inhibitory control, and attention shifting for young children. These tasks have been associated with children's school readiness. The EF Touch assessment includes the use of a touch-screen laptop and an external monitor. The child responds via the touch-screen laptop and the assessor reads the task directions from the external laptop. Assessors may also wish to use a USB powered mouse, keyboard, or number pad.

There are eight tasks in the EF Touch program, to complete administration of all of the tasks takes between 45-60 minutes. Each individual task takes approximately 5 minutes to administer. Often not all of the tasks are administered together due to the age of the child, the tasks being administered as part of a larger testing protocol, or limitations in the time allotted for data collection. For information on alternative short forms administrations please see Willoughby, Pek, & Blair, 2013. The tasks are as follows:

Arrows

This is an inhibitory control task, measuring children's ability to override a dominant response. It is adapted from a task developed by Gerardi-Caulton (2000). In this game, children are asked to follow the rule of "touching the button in whichever direction the arrow is pointing". On initial trials the arrow is located on the same side of the screen as the button that should be touched. This becomes the child's dominant response. However, in more difficult items the direction the arrow is pointing is in direct conflict with the child's dominant response (e.g., the button is located on the opposite side of the screen as the arrow).

During the pretest phase items are provided that teach children the task (“touch this [left] button when arrows points this [left] way and touch this [right] button when arrows point this [right] way”). During the administration items, arrows are depicted laterally, with the left pointing arrows always appearing on the left side of the screen (“above” the left button) and the right pointing arrows always appearing on the right side on the screen (“above” the right button). Or, arrows are depicted contra-laterally, with the left pointing arrows appearing on the right side of the screen and the right pointing arrows appearing on the left side of the screen. Items presented contra-laterally require inhibitory control from the previously established pre-potent response in order to be answered correctly (spatial location is no longer informative). Children are encouraged and prompted (but not required) to touch the left button with their left hand and right button with their right hand. At the end of the task the task administrator is prompted with a question regarding children’s hand preference. There are 36 items. This task works well with all age groups.

Houses

This task measures working memory and is based on work from Kane & Engle, 2003. Working memory involves holding information in short-term storage and attending to one item while overcoming interference from the other. In this task, children are presented with pictures of houses with animals and colors in them. The child is asked to name the type of animal and color in each of the houses and then, after a short delay, the house is presented again - but this time the house is empty. The child is asked to recall only one piece of information (e.g., either the color or the animal that was in the house). The task requires children to perform the operation of naming and holding in mind two pieces of information simultaneously and to activate one while overcoming interference occurring from the other. The task becomes more difficult as the

number of houses increases. The task is made still more difficult by binding the stimulus properties, that is, coloring the animal and asking the child to name the color or the animal.

In the pretest phase, it is established that children can name both the colors and the animals in the task. Children then receive three 1-house trial, three 2-house trials, and three 3-house trials. There are 18 items. This task works well with 4- and 5-year-olds. It can be presented successfully to 3-year-olds, but it will be challenging. We do not recommend using this task in combination with the other working memory tasks for 3-year-olds.

Silly Sounds Game (SSG)

This task measures inhibitory control and is a simple Stroop-like task in which the child must overcome a highly learned response (derived from Gerstadt, Hong, and Diamond's (1994) Day-Night task). In this task, the child is presented with side-by-side pictures of a cat and a dog (in random order). For each trial, either a dog bark or cat meow sound is played. Children are required to touch the picture of the cat when they hear the dog barking and to touch the picture of the dog when they hear the cat meow. The task becomes more difficult with time, as children may forget to override their dominate response.

During the pretest phase, the experimenter introduces the idea that, in the Silly Sounds game, dogs make the sounds of cats and vice versa. Scripted coaching and elaboration is provided. The verbal prompts (i.e., "what sound does this animal make in the Silly Sounds game?") are discontinued after the first 8 items (the experimenter just lets the program run). A total of 17 items are presented. This task works well with all age groups.

Something's the Same (STS)

This task measures attention shifting and requires children to use flexible thinking. It was adapted from the Flexible Item Selection Task developed by Jacques and Zelazo (2001). For the initial trials in the task, children are presented with two pictures (animals, flowers, etc.) that are similar along a single dimension of color, shape, or size. Initially, the child is explicitly told how two of the pictures are the same in some way. Then, the child is then presented with a third picture alongside the original two and asked to state how the new picture is similar to one of the original pictures (e.g., if the first two pictures were similar along the dimension of shape, the third card would be similar to one of the first two along the dimension of color or size). This task requires the child to shift his/her attention from the initial dimension of similarity to a new dimension of similarity. In the most difficult items all of the pictures are presented at once and children are prompted to identify both dimensions of similarity.

During the pretest phase it is established that children understand color and size (i.e., big and small). Items 1-20 are presented in the two-then-three picture manner. Items 20-30 are presented in a three picture manner and children can now match on the concept of category. There are 30 items. This task works well with all age groups.

Pig

This task measures inhibitory motor control and is a standard go no-go task (Durston, Thomas, Yang, Ulug, Zimmerman, & Casey, 2002). Children are presented with a large green button on the screen that makes a “popping” sound when it is touched. Children are instructed to touch the button every time that they see an animal (the ‘go’ response) except when that animal is a pig (the ‘no-go’ response). No-go responses vary in difficulty depending on how many go responses preceded them.

In the pretest phase, children are asked to identify all of the animals. During administration items, the task is presented in varying numbers of go trials prior to each no-go trial, including, in standard order, 1-go, 3-go, 3-go, 5-go, 1-go, 5-go, 7-go and 7-go. There are 40 items. This task works well with all age groups.

Pick the Picture (PTP)

This task measures working memory and is adapted from a self-ordered pointing task (Cragg & Nation, 2007; Petrides & Milner, 1982). Children are presented with a series of progressive larger (2, 3, 4, 6) sets of pictures. For each set, the child is initially instructed to touch any picture of their choice. Each set of pictures is repeatedly presented, with the location of the pictures changing in a randomized order. Children are instructed to continue to touch a new picture in the set so that “all of the pictures get a turn”. For example, in the 2-picture condition, they might see a screen with pictures of an apple and a dog. On the first screen, they might touch (pick) the picture of the apple. On the second screen, the same two pictures are presented but in a different order. Children are required to recall which picture they previously touched and to touch a new picture—in this example the dog. The task requires working memory because children have to remember which pictures in each item set that they have already touched.

The arrangement of pictures within each set is randomly changed across trials (including some trials not changing) so that spatial location is not informative. There are 32 items. This task works well with 4- and 5-year-olds. It can be presented successfully to 3-year-olds, but it will be challenging. We do not recommend using this task in combination with the other working memory tasks for 3-year-olds.

Farmer

This task measures children's visual spatial working memory ability and is derived from a task developed by Nutely and colleagues (2010). In the game, children are presented with a 4 by 4 grid of squares depicted as a farmer's fields. One of the farmer's animals gets lose and the child is instructed to help the farmer remember which fields that the animal has "walked in" to help the farmer take him home. The child is shown a number of sequentially highlighted fields and then asked to touch them in the same order that the animal walked in them (i.e., the order in which they were highlighted). The items become increasingly difficult as the number of fields that an animal walks in increases and children are asked to recall longer strings of fields.

There are 36 items. This task works with 4- and 5-years-olds; it is not designed for the assessment of 3-year-olds.

Bubbles

This task is a measure of children's speed of processing. Children are asked to touch pictures of blue bubbles, as they appear on the screen, as fast as they can. There are 30 items. This task works well with all age groups.

Chapter Five

Administering the EF Touch Battery

Starting the Program

- Set up the laptop at a 90 degree angle so that the child can respond on the laptop but not be distracted by the external monitor. Consider the configuration of the touch screen; you may want the laptop to be in tent mode. You can use Ctrl+Alt+Up arrow keys as a shortcut to change the screen orientation.
- If using a touch monitor ensure that all of the cables are correctly connected to the laptop.
- Double click the EF Touch program icon on the desktop to launch the program. Two monitors must be connected for the program to launch.
- Once the program is started the EF Touch v0.12 box will pop up. There will be several buttons on the left side of the box to navigate the tasks.
- Next, click on the **Add Child** button.
- This brings up the child information box. Enter data into the child ID, DOB, and language fields. Data in the visit field is optional. When finished click on **OK**.
- The EF Touch program has multiple tasks and the eight tasks are displayed at the top of the dialog box.
- Administer tasks across the task bar, not up and down.
- Note: there are several advanced options under the **Setup** button. Here you can select the tasks that you would like viewable, edit input ranges, and manage the format of data output files.
- Additional help can be found under the **Docs** button.

Starting the Assessment

- Administer the Training Task first. Most children have never done this type of assessment before, and this gives them the opportunity to become familiar with the touch-screen monitor. This task is also an opportunity for you to 'teach' colors if the child seems shy or unsure. This is not a scored task. Place your cursor in the training box for your id # and "Double click" or click on the **Run Task** button to open the task.
- A word about touching. Some children will need assistance with learning how to *touch* the touch screen; that is with the appropriate amount of force and quickness so that their touch registers. The training task is the time to help children figure this out.
- Managing touching behaviors. Many children will want to touch the touch screen at inappropriate times (e.g., all the time or at times when the screen will not respond to their touch). It is important to establish where children should keep their hands (e.g., on the table or in their laps) during the tasks and when they should touch the screen. During some of the games, you may need to remind children to "Don't touch now" or "Wait for the picture" before they can touch the screen.
- Enter your Administrator ID to begin the task.
- Bracketed text indicates that the assessor does something and this is not read to the child.
- Words are on the screen are to be read to the child. Read exactly as shown on the screen.
- The training items require you to provide correction or give praise. If the corrections or praise are not scripted, you may ad-lib those phrases. Corrections should make clear to the child what the expected response was. Examples: "I asked you to touch the yellow dot and you didn't touch the yellow dot. See, I'll touch the yellow dot." "Good job."

- To advance, touch the space bar. You'll typically hear a sound.
- The top right of the screen shows the words "Training" and how many items are left. Once you begin the actual tasks, you can also see how many items are left, example: "12 out of 24."
- The computer does record "reaction time" on the timed items.
- The computer does all of the scoring, including determining if children pass the training items to proceed onto the game.
- At the end of each task you will be required to complete a rating of the administration of the task. There will be a "Data Quality" box with a three point scale for the assessor to rate the administration. This allows you to rate your impression of the quality of the data. For example, were there serious concerns during the assessment (e.g., child under the table and not focused at all on the task; there was a fire drill during the task)? The rating allows you to provide your impression about aspects of the assessment that we would not know just by looking at the data. In other words, this is not another rating of the number of items that the child got correct.
- Tasks are designed to be "modular" so that one task can be given at a time. If a child needs a break (bathroom, stretch), you may stop at the end of one task, allow the child to have a break, then come back and start the next task. Ideally, we do not want to take a lot of time between tasks, but sometimes stretching, jumping jacks, or a water break may help the child refocus on the task.

The Tasks

Arrows: Measures inhibitory control – the ability to override a dominant response.

- Child’s hands can sit on the table or his/her lap
- Read the task instruction exactly as scripted on the screen.
- Praise or correction can be as follows:

  “I’d like you to use this hand on this side” (Point to or touch the child’s hand to show which hand should touch the dot). “Good job touching the button quickly.”
- The training is designed to teach the child the task. Once the actual task is started, the only correction allowed is to refocus the child on the task “Remember we are playing a game on the computer.” *Prompt says do not use further instructions within the test items.*
- It’s ok if the child does not touch a button. It will be recorded as no attempts.
- The speed will feel slow to adults, but when you’re working with 3 year olds, it takes a little longer for them to process information.
- If children ask questions when the test has started, say, “Just go ahead and go along with the game. We’re not going to stop the game.”
- To stop the task – In extreme cases, you may need to stop the task, e.g., fire drill, bathroom emergency, etc. In that case, you may click on “X-Quit” at the top right to end the task.
- Complete hand dominance question and data quality rating at the end of the task.

Houses: This is a task of working memory – holding information in short-term memory and using that information.

- For training items, when you need to provide a correction, say something like “No, that’s the X (fish, etc.) in that house”

- Pause and Delay – There is a slight pause and delay in this task. The task intentionally imposes a delay which makes the task more difficult. This isn't a computer problem.
- Make sure you read all the text on the page.
- There's a choice for "other" when a child responds with an animal that is not present or with a bizarre response. This is okay and does not require the assessor to force another response from the child.
- Highlighted houses – Notice that in multiple house trials the first house is highlighted. You are asking about the highlighted house, when you advance to the next house it will become highlighted.
- The assessor does not have to remember the animals in the houses. The computer knows the correct animals. Just enter what the children say. Children frequently say the same thing over and over again.
- Complete the data quality rating at the end of the task.

Silly Sounds Game: An inhibitory control task, requiring the child to override a dominant response.

- Child must pass the training in order to advance to the test items. If a child cannot correctly identify dog and cat once each for the opposite sound, the task will cycle back to the training for a second time. If the child cannot complete the training items again, the task will end.
- Correction for Silly Sounds – "Remember, this a Silly Sounds game; we're being very silly. When you hear the woof sound, touch the cat. When you hear the meow sound, touch the dog."

- Children sometimes respond, "No, dogs bark and cats meow." The assessor can say, "You're exactly right, but in the Silly Sounds game, cats bark and dogs meow. Can you be silly with me?"
- Once you get out of training mode, the computer goes on its own.
- Do not provide any training or corrections during the actual task.
- Complete the data quality rating at the end of the task.

Something's the Same: This measures attention shifting which is the ability to represent an idea that can be thought of in different ways.

- In this task, you will not see at prompt at the top of the screen to indicate that the training has ended. It will go directly into test mode. Follow screen script exactly so that you'll know when NOT to provide a correction.
- You can assist children if they are unsure about their colors during the training items.
- This is not a speeded item task.
- If the child responds, "None of them are the same." The assessor can prompt with, "You can take a guess" or "Just do your best" or "Give a try" and then "which one is the same as 'X.'" If child responds a second time with, "None of them are the same," the assessor enters "Child did not respond".
- If child touches the new picture on the right, the screen will not respond. You can say, "Remember, which one of these (point to two pictures on the left) is the same as this new one (point to picture on the right)?"
- This task does not automatically advance to the next screen. The assessor must advance the screen by clicking enter or the space bar.

- Starting at item #20, slightly more abstract items are shown. Here kids might say, "None are the same." Say "Do your best" or "Make a guess," but, don't say this every time.

Pig: This is an inhibitory motor control task

- In this task you can help children identity the different animal during the training items.
- Once you get out of training mode, the computer goes on its own.
- This is a speeded item task
- Once the task begins do not provide feedback, but you may remind the child to "keep playing the game" if they get distracted or look away from the screen.
- Complete the data quality rating at the end of the task.

Pick the Picture: This is a working memory task– holding information short-term and using that information.

- This is not a speeded item task
- Encourage children to "take a guess" if they do not remember which picture has not had a turn.
- Children frequently pick the same picture over and over again.
- This task does not automatically advance to the next trial. The assessor must advance to the next trial by clicking the space bar or the **Next** button.
- Complete the data quality rating at the end of the task.

Farmer: This is a task is of visual spatial working memory.

- This task will be difficult for young children and is not recommended for 3-yr-old children.
- Remember the assessor needs to watch which fields the animal walks in during the training items to provide corrections for the child.
- In the last training item (#3), the animal walks in the same field twice.
- It is critical that children are paying attention when the animals are presented (get into the fields). You may need to remind children to watch when the animals are presented.
- Complete the data quality rating at the end of the task.

Bubbles: This measures simple reaction time.

- There is no training for this task.
- Children generally enjoy this task and it can be used as a ‘break’ when a child gets frustrated.

Chapter Six

Setting Up EF Touch Battery Equipment

Connecting External Monitor to the Laptop

- There is one USB cable –that's how the laptop and monitor communicate with each other- that is plugged into the back of the external monitor and into a USB slot on the laptop.

Connecting Planar Monitor to the Laptop

- There are four cables to connect the laptop and Planar: VGA, USB, speaker and power adapter. These four cables are connected in the back of the Planar monitor.

Troubleshooting

- If you turn on the laptop and you get a message on the screen indicating a "dual" screen/monitor is present (e.g., the laptop screen shows on the touch screen) do the follow:
  - Right click on the background screen.
  - Click on screen resolution.
  - Under the *multiple displays* option select "Extend these displays" from the drop down menu (it now recognizes the monitor as a single monitor).
  - Click on Ok.
- If using a Planar monitor make sure that your drivers are current at this website http://apps.planar.com/downloads/touchscreen/

- If using a Planar monitor you may need to run the MultiMonitor Manager program. This program will appear as an icon on your desktop after installing the Planar software. To open double click on the MultiMonitor Manager icon. Confirm that the Planar monitor is designated as your touch monitor. To do this make sure that the Planar monitor is assigned as the number 2 screen and highlighted by the bright blue color. Conversely, your laptop should be highlighted by the dark blue color.

Chapter Seven

EF Touch Battery Scoring

The variables that will be the most useful to you are the PrpCor ones. You will note this score is provided for each individual game, for example there is Arr_PrpCor variable for the proportion correct in Arrows.

This score is a portion from 0 to 1, zero indicating no items correct and 1 indicating all items were correct. You may also want to consider the PrpCmp variables when interpreting the portion correct. These variables tells you what portion of item were completed, again this is for each individual task, so there is Arr_PrpCmp for the portion of items completed in Arrows.

There are also mean reaction time scores for some of the tasks, Mean_ms (example: Pig_Mean_ms is the mean score for Pig) and scores for each unique item (example: Pig_2 is the score for item 2 in the Pig task).

For all of the games the minimum is zero, but the maximum varies by game.

- Arrows: 4 seconds
- Silly Sounds Game (SSG) and Pig: 3.5 seconds
- Any other timed tasks: 5 seconds

Variable Names

ChildID= Child identification number as determined by researcher

Visit=Label determined by researcher

DOB=Child date of birth in mm/dd/yyyy format

Language=Options on English, Spanish or Chinese

Version=Current version is 0.12

TasksAttempted=Number of tasks attempted by the child out of 8

TasksFailed=Number of tasks the child failed the training items

TasksAdministered=Number of tasks administered by the assessor – includes tasks that child may have failed the training items

OrderAttempted=Order in which the tasks were administered

AgeFirstAdministration=Calculated child age

Tr_Date=Date Training task was administered

Tr_BeginTime=Time at which the Training task was administered

Tr_ElaspedTime=How long it took for the Training task to be administered

Tr_Status=The status of the Training task with values of empty, indicating the task was not attempted, 0, indicating the training was failed, 1, the indicating the task was not completed, or, 2, indicating the task was completed

Tr_NumTrFail=Number of items failed in the Training task

Arr_AdminID= ID of assessor who administered Arrows task

Arr_Date= Date Arrows task was administered

Arr_BeginTime= Time at which the Arrows task was administered

Arr_ElapsedTime= How long it took for the Arrows task to be administered

Arr_Status=The status of the Arrows task with values of empty, indicating the task was not attempted, 0, indicating the training was failed, 1, the indicating the task was not completed, or, 2, indicating the task was completed

Arr_ NumTrFail= Number of training items failed in the Arrows task

ArrQuality=Quality of data as rated by assessor on 3-point scale

Arr_NumCmp=The number of items completed in the Arrows task

Arr_HandPref=The child's hand preference during the Arrows task, 1, indicating the use of only one single dominate hand, or, 2, indicating use of both hands.

Arr_PrpCmp=Proportion of items completed in the Arrows task

ArrPrpCor=Proportion of items correct in the Arrows task

Arr_Mean_ms=Mean reaction time in milliseconds for Arrows task

Arr_StdDev_ms= Standard Deviation for the mean reaction time of the Arrows task

Arr_PrpCmpCongru= Proportion of congruent items completed in the Arrows task

Arr_PrpCorCongru= Proportion of congruent items correct in the Arrows task

Arr_MeanCongru_ms= Mean reaction time in milliseconds for congruent items on the Arrows task

Arr_StdDevCongru_ms= Standard Deviation for the mean reaction time of congruent items on the Arrows task

Arr_PrpCmpSwitch= Proportion of incongruent items completed in the Arrows task

Arr_PrpCorSwitch= Proportion of incongruent items correct in the Arrows task

Arr_MeanSwitch_ms= Mean reaction time in milliseconds for incongruent items on the Arrows task

Arr_StdDevSwitch_ms= Standard Deviation for the mean reaction time of congruent items on the Arrows task

*****The same variable naming convention is used across all of the tasks. The prefixes are as follows:**

Pig=Pig, Hse=House, PTP=Pick the Picture, SSG=Silly Sounds Game, STS=Something's the Same, Frm=Farmer, Bbl=Bubbles

Tasks Specific Variables

Pig_PrpCmpNo=Proportion complete on Pig items of the Pig task

Pig_PrpCorNo=Proportion correct on Pig items of the Pig task

Pig_MeanNo_ms= Mean reaction time in milliseconds for Pig items on the Pig task

Pig_StdDevNo_ms= Standard Deviation for the mean reaction time of Pig items on the Pig task

Pig_PrpCmpGo=Proportion complete on non-Pig items of the Pig task

Pig_PrpCorGo=Proportion correct on non-Pig items of the Pig task

Pig_MeanGo_ms= Mean reaction time in milliseconds for non- Pig items on the Pig task

Pig_StdDevGo_ms= Standard Deviation for the mean reaction time of non-Pig items on the Pig task
